# Convolution- and Attention-Based Neural Network for Automated Sleep Stage Classification

**DOI:** 10.3390/ijerph17114152

**Published:** 2020-06-10

**Authors:** Tianqi Zhu, Wei Luo, Feng Yu

**Affiliations:** College of Biomedical Engineering and Instrument Science, Zhejiang University, Hangzhou 310027, China; terryzhu@zju.edu.cn (T.Z.); willi4m@zju.edu.cn (W.L.)

**Keywords:** sleep stage classification, convolutional neural network, attention mechanism

## Abstract

Analyzing polysomnography (PSG) is an effective method for evaluating sleep health; however, the sleep stage scoring required for PSG analysis is a time-consuming effort for an experienced medical expert. When scoring sleep epochs, experts pay attention to find specific signal characteristics (e.g., K-complexes and spindles), and sometimes need to integrate information from preceding and subsequent epochs in order to make a decision. To imitate this process and to build a more interpretable deep learning model, we propose a neural network based on a convolutional network (CNN) and attention mechanism to perform automatic sleep staging. The CNN learns local signal characteristics, and the attention mechanism excels in learning inter- and intra-epoch features. In experiments on the public sleep-edf and sleep-edfx databases with different training and testing set partitioning methods, our model achieved overall accuracies of 93.7% and 82.8%, and macro-average F1-scores of 84.5 and 77.8, respectively, outperforming recently reported machine learning-based methods.

## 1. Introduction

**Figure 1:**
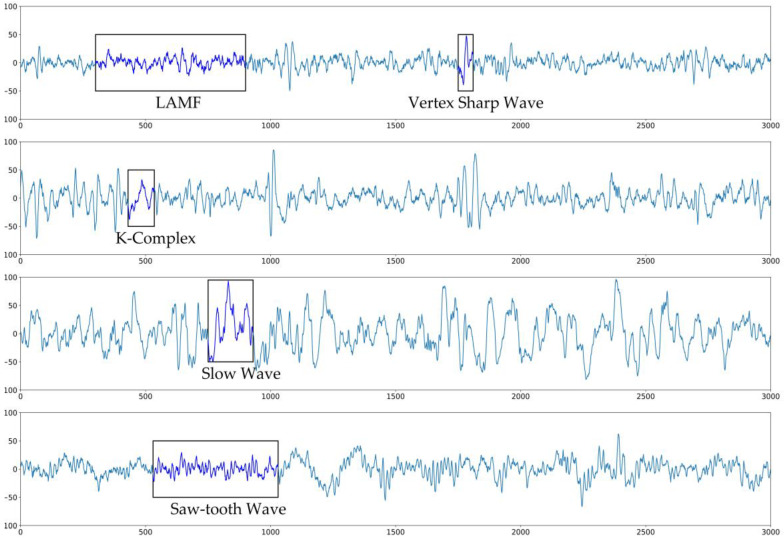
Examples of the characteristic electroencephalogram (EEG) signal features of sleep stages (from top to bottom): stage N1 with low amplitude mixed frequency (LAMF) and vertex sharp waves; stage N2 with K-complexes; stage N3 with slow waves; and rapid eye movement (REM) sleep stage with saw-tooth waves.

Sleep is an essential human activity that occupies one-third of people’s lives. Long periods of unhealthy sleep can lead to various diseases [1,2]. Medical experts assess five components of sleep health: duration, continuity, timing, alertness, and quality [3]. Most of these indicators can be obtained via polysomnography (PSG) analysis. The acquisition and analysis process of PSG is as follows. First, multiple sensors placed on the patient record physiological signals—producing an electroencephalogram (EEG), electrooculogram (EOG), electrocardiogram (ECG), and electromyogram (EMG)—during sleep. Second, these signals are split into 30-s epochs that are classified by sleep state: wake (W), rapid eye movement (REM), non-REM stage 1 (N1), non-REM stage 2 (N2), non-REM stage 3 (N3), and non-REM stage 4 (N4), as defined by the Rechtschaffen and Kales Manual (R&K) [4]; or by merging stage N4 into stage N3, as defined by the American Academy of Sleep Medicine Manual (AASM) [5]. Third, the scorer notes spontaneous arousals, cardiac arrhythmias, and respiratory events. In this process, the second step is both crucial and time-consuming [6]. It requires that an experienced medical expert observe each PSG epoch to look for its characteristic features and assign it to the correct sleep stage. Figure 1 shows some examples. This labor-intensive process limits the efficiency of PSG analysis. With extensive researches of machine learning methods in biomedicine [7,8,9,10,11], many researchers have proposed a series of machine learning-based algorithms to carry out computer-aided, or even fully automated, sleep stage classification [12,13,14,15].

In recent years, automated sleep stage classification research has focused on two machine learning approaches [6]: traditional machine learning methods and deep learning-based methods. Traditional machine learning methods combine manually chosen representative signal features and machine learning models to classify sleep stages. For example, Liang et al. [16] first proposed the use of multiscale entropy as a signal feature, and employed an autoregressive model for classification. Tsinalis et al. [17] extracted 557 features in the time-frequency domains of EEG signals as input to a stacked sparse autoencoder model, and achieved 78.9% accuracy on the sleep-edf [18] database. A study by Hassan et al. [19] handled a signal that needed to be decomposed into several sub-bands, using the Tunable-Q wavelet transform. Classification based on a bootstrap aggregating model was then implemented based on the statistical characteristics of the sub-bands. Jiang et al. [20] divided sleep stage classification into three steps: feature extraction based on multimodal decomposition, classification using a random forest, and result refinement based on sleep stage transition rules using a hidden Markov model. The refinement process was particularly suited to improving the classification accuracy of stage N1.

In deep learning models, feature extraction is automatically realized by a deep neural network model [21,22], enabling end-to-end automated sleep stage classification. Deep learning-based methods mainly use convolutional neural networks (CNNs) [23], recurrent neural networks (RNN), or a combination of the two. CNNs have a strong capacity to learn shift-invariant features, and have already achieved great success in the field of computer vision. ResNet is a powerful architecture in image classification. Andreotti et al. [24] first employed a modified ResNet with 34 layers to realize automatic sleep stage classification. Yildirm et al. [25] developed a one-dimensional CNN that used raw PSG signals as input, and achieved 91% accuracy on the sleep-edf dataset. Phan et al. [26] proposed a two-dimensional CNN-based model. Their method obtains a spectral map using a short-time Fourier transform of the raw PSG and employs a classification process similar to that used for natural images. However, labeling an epoch, whether using the R&K guideline or the AASM, sometimes requires combining its data with information from the previous and following epochs. RNNs are often used to deal with problems, like this one, that include time dimension information. Among several RNN methods, long short-term memory (LSTM) [27] is the most widely used, and can competently deal with long-term temporal dependence. Michielli et al. [28] used a two-level LSTM structure to classify EEG signals, which can effectively improve the classification performance of the N1 stage. The method of combining a CNN and LSTM was first proposed by Supratak et al. [29]. The model used the CNN module to extract epoch-wise features, and then used bidirectional LSTM to extract sequence features to classify epochs. 

In this study, we propose a neural network model based on a CNN and an attention mechanism [30] for automated sleep stage classification, using a single-channel raw EEG signal. The main contributions of this work are as follows:

- A neural network based on convolution and attention mechanism is built. The network uses a CNN to extract local signal features and multilayer attention networks to learn intra- and inter-epoch features. The recursive architecture is completely deprecated in our model.
- For the unbalanced dataset, the proposed method uses a weighted loss function during training to improve model performance on minority classes.
- The model outperforms other methods on sleep-edf and sleep-edfx datasets utilizing various training and testing set partitioning methods without changing the model’s structure or any of its parameters.

## 2. Materials and Methods 

### 2.1. Dataset and Preprocessing

In this study, the sleep-edf and sleep-edf expanded (sleep-edfx) databases were used to evaluate our model’s performance. These two public datasets are published on PhysioNet [31] and are widely used for research on automatic sleep stage classification algorithms. There are eight sleep records in the sleep-edf database, four from healthy subjects and four from subjects with sleep disorders. Sleep-edfx contains 197 records of 61 healthy individuals and 20 individuals with sleep disorders. Each record is a whole-night PSG recording containing EEG Fpz-Cz, EEG Pz-Oz, EOG, EMG, and manual staging records. We compared our results with those of state-of-the-art machine learning-based sleep staging methods [19,25,26,29] on the complete sleep-edf database and on the first 20 healthy individual records (subjects 0–19) from the sleep-edfx database. For each record in the sleep-edfx dataset, 30 min of wake stage data were retrained from before the first sleep epoch and from after the final sleep epoch. As per the latest AASM manual, we merged stages N3 and N4 into a single slow-wave stage. The distribution of the processed data is shown in Table 1.

**Figure 2:**
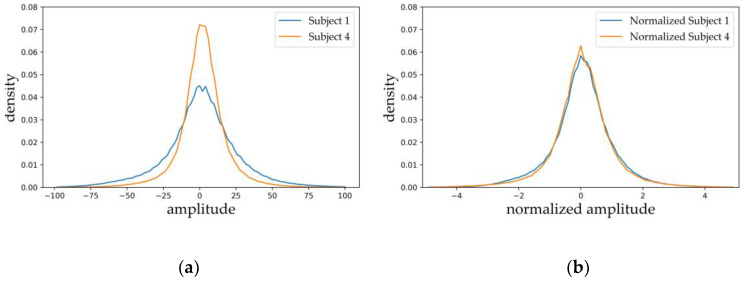
EEG signal amplitude distribution: (**a**) raw data; (**b**) normalized data.

The model used the Fpz-Cz channel as input. Due to differences between individuals, collection equipment, and environments, the resulting data distributions also have distinct differences (Figure 2a) that make the model difficult to train. In order to make the training more stable, we performed *z*-score normalization on the data from each individual. The normalized data distribution is shown in Figure 2b.

**Figure 3:**
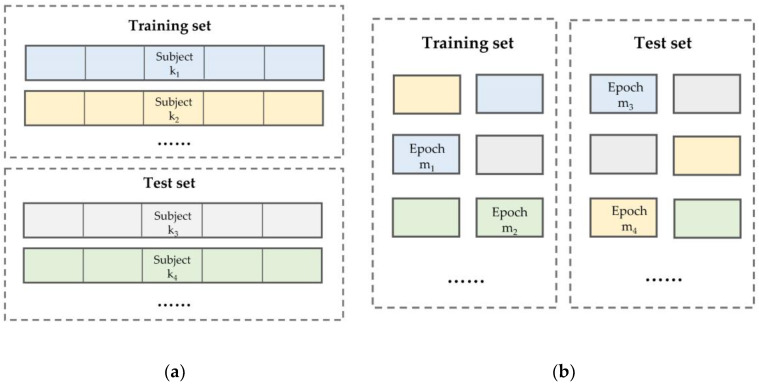
Two training and testing set partitioning methods: (**a**) subject-wise; (**b**) epoch-wise.

Machine learning algorithms require independent training and test sets for model training and performance evaluation. There are two types of training data partitioning methods for clinical data—subject-wise and record-wise (called epoch-wise in our work, see Figure 3); these are also called independent and non-independent methods, respectively, in some papers [20,29]. This article uses the epoch-wise method on the sleep-edf database and the subject-wise method on the sleep-edfx database. In the epoch-wise method, the dataset is shuffled before partitioning.

### 2.2. Model Architecture

**Figure 4:**
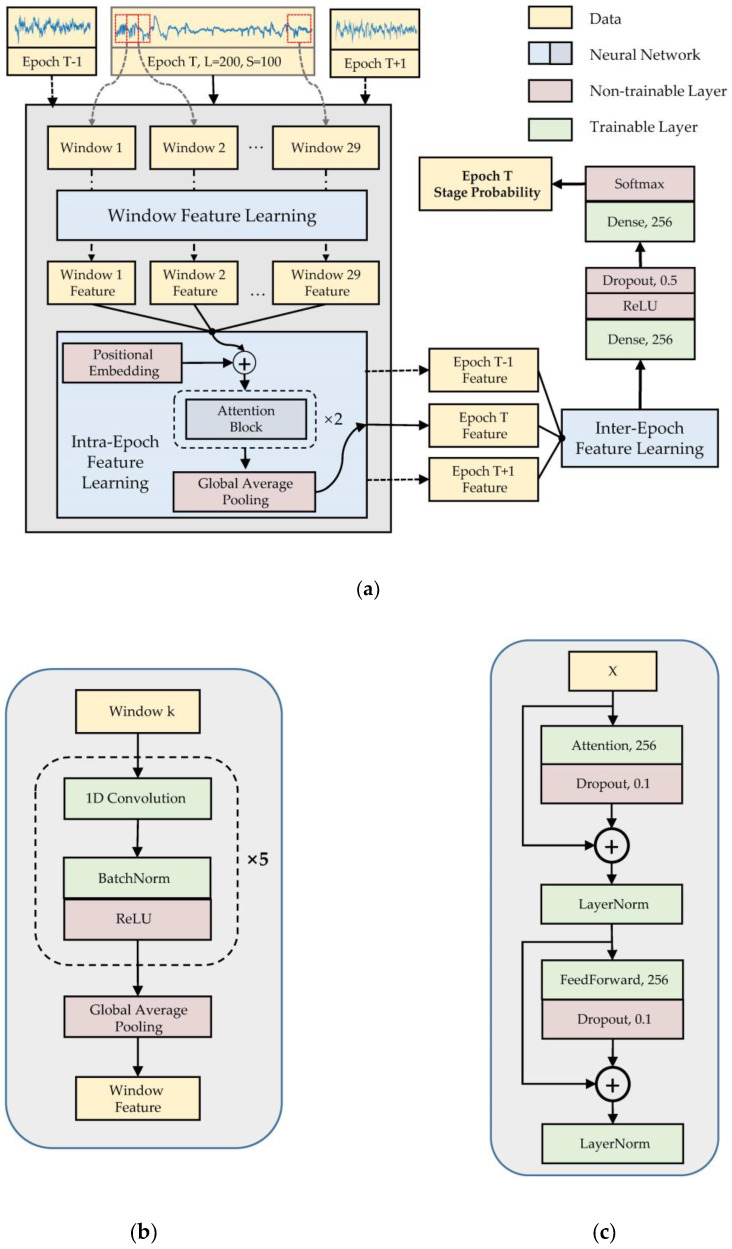
Model architecture: (**a**) whole model; (**b**) window feature learning; (**c**) attention block.

Our model has three components: window feature learning, intra-epoch feature learning, and inter-epoch feature learning (Figure 4). The model inputs multiple signal windows to the window feature learning module in parallel. The module uses a deep CNN to construct a feature vector for each window. The intra-epoch feature learning is based on the self-attention mechanism to obtain the weight of each signal window in an epoch, and then adds windows features by these weights to obtain the epoch feature. The window feature is updated in this part via a feed-forward layer [30]. The inter-epoch feature learning component also uses the self-attention mechanism to learn the temporal dependency between the current epoch and the adjacent epochs, to obtain more representative features for the current epoch.

Many features used in sleep staging are short-term, such as K-complexes and spindles. The duration of these characteristics is usually only 0.5–1.5 s. Some overall features, such as LAMF, can also be obtained by synthesizing short-term features. Therefore, in order to more effectively capture short-term features, we divided the epoch into multiple windows and used CNNs to extract the features of the window. To avoid truncating a feature between two windows, some overlap was left between the windows. In our experiment, the window length is 200 and the overlap length is 100, so each epoch has 29 windows. The window feature learning model is detailed in Figure 4b. This component consists of five convolution blocks and a global average pooling (GAP) layer [32]. Each convolution block contains a one-dimensional convolutional layer, batch normalization layer [33], and rectified linear unit (ReLU) activation layer [34]. The batch normalization parameters in the module are momentum, set to 0.99, and epsilon, set to 0.001. The parameters of the convolution layer are shown in Table 2.

Intra- and inter-epoch feature learning have the same model structure, which consists of positional embedding [29], two identical attention blocks, and one GAP layer. They differ in their inputs: intra-epoch feature learning uses window features with shape (29, 256) and inter-epoch feature learning uses epoch features with shape (3, 256). The attention module has a structure similar to the Transformer encoder [29], as shown in Figure 4c. Assuming that the input to the attention and feed-forward layers is $X={\left(x_{1},x_{2},\ldots ,x_{n}\right)}^{T}$, $x_{i}\in {\mathbb{R}}^{1\times L}$, the operations of these two layers can be defined as follows:

(1) $$Q={\mathrm{XW}}_{a}+b_{a}$$

(2) $$\alpha =\mathrm{Softmax}\left(\mathrm{Tan}h\left({\mathrm{QX}}^{T}\right)\right)$$

(3) Attention(X)=αX

(4) $$\mathrm{FeedForward}\left(X\right)=\max\left(0,{\mathrm{XW}}_{f1}+b_{f1}\right)W_{f2}+b_{f2}$$

where $W_{a},{\;W}_{f1},{\;W}_{f2}\in {\mathbb{R}}^{d\times d},{\;b}_{a},{\;b}_{f1},{\;b}_{f2}\in {\mathbb{R}}^{1\times d}$, weight dimension d is 256, and the dropout [35] and layer normalization [36] parameters in this component are 0.1 and 0.001, respectively.

After the previous three components, we finally obtained the feature vector of the current epoch with shape (1, 256). The model uses two fully connected layers as the classifier and will output each stage class probability of the current epoch. The first fully connected layer contains the ReLU and dropout layers. The second fully connected layer connects to the softmax layer, which normalizes the output probability.

### 2.3. Training and Testing

#### 2.3.1. Training Parameters

To reduce the impact of class imbalances and improve the model’s accuracy in identifying minority classes, we used a class weighted cross-entropy loss function in training, defined as:

(5) $$\mathrm{loss}=\beta_{i}*y_{i}*\log\left(y_{i}^{\mathrm{pred}}\right).$$

Weight β_i_ corresponds to real category y_i_. In the sleep-edf experiment, the weights of the wake, N1, N2, N3, and REM stages were 1.0, 4.0, 2.0, 2.0, and 2.0, respectively; in the sleep-edfx experiment, they were 2.0, 4.0, 2.0, 1.0, and 2.0. We did not completely rely on the number of samples in each category to set the parameters; we simply set the majority category to 1.0, intermediate categories to 2.0, and the minority category to 4.0 to avoid overfitting in training. We used the Adam [37] optimizer combined with a LookAhead mechanism [38], in which the initial learning rate was 1e−4, the learning rate decay was 2e−4, and the gradient clip value was 0.1.

#### 2.3.2. Testing Method

For the two datasets, which used different partitioning methods, we used different training and testing methods. On the sleep-edf dataset, we divided the dataset into 70% training set and 30% test set epoch-wise. The training set was trained with 100 epochs (the number of iterations on the entire training set, which is different from sleep epochs) and the model performance was evaluated on the test set. On the sleep-edfx dataset, which used the subject-wise partitioning method, we used the leave-one-out method. That is, each training process used 19 subjects as a training set and tested the remaining subject; this process was repeated 20 times to evaluate the model’s performance on the entire dataset. Since there are more samples in the sleep-edfx dataset, each training consisted of only 35 epochs.

Since we do not use a validation dataset, the early stopping strategy was not used during training. We used the ensemble method to improve the model’s generalizability and stability. The principle underlying this method is that ensemble outputs are obtained by using multiple models to infer the same input to get a final output, as shown in Equation (6), where P_i_ (X_t_) is the stage probability vector of model i for the input at time t, and y_t_ is the final output stage. Here we save the parameters of the last five epochs of the model during training to obtain multiple models.

(6) $$y_{t}=\mathrm{argmax}\left(\prod_{i\in \Lambda }P_{i}\left(X_{t}\right)\right)$$

#### 2.3.3. Performance Metrics

To comprehensively evaluate the model’s performance, we evaluated it per category and overall. For each category, we calculated the precision, recall, and F1-score of the model, where the F1-score is defined as in Equation (7). For the overall evaluation, we used the accuracy to obtain an intuitive understanding of the model’s performance on the entire dataset. However, because the distribution of each stage in the dataset is uneven, overall accuracy cannot reflect the model’s true performance. For example, imagine a dataset with two categories, A and B, in which the proportion of A is 99%. Then, even if the model incorrectly classifies all B as A, the model’s overall accuracy is still 99%. The negative and positive proportions of some diseases in the population exist in similar proportions, meaning that we cannot accept such classification results for clinical use. To better reflect the model’s performance on imbalanced datasets, we used the macro average F1-score (MF1) to evaluate it. MF1 is defined in Equation (8), with C = 5 to represent the number of sleep stage categories.

(7) $$F1_{i}=\left(\mathrm{precision}+\mathrm{recall}\right)/\left(2*\mathrm{precision}*\mathrm{recall}\right)$$

(8) $$\mathrm{MF}1=\Sigma_{i=1}^{C}F1_{i}/C$$

## 3. Results

### 3.1. Model Performance

Table 3 shows the performance of our model on the sleep-edf dataset with epoch-wise partitioning. Its overall accuracy is 93.7%, and its MF1 is 84.5. Table 4 shows the model’s performance on the sleep-edfx dataset with subject-wise partitioning. Its overall accuracy is 82.8%, and its MF1 is 77.8, which reached the inter-rater agreement (83%) among stages [39]. In these two experiments, the accuracy of the wake, N2, N3, and REM stages were similar and relatively reliable; their F1-scores are all greater than 80. In contrast, the classification accuracy of stage N1 is poor, significantly lower than that of the other categories. Due to the small number of stage N1 samples, this problem is not reflected in the overall accuracy; however, the model’s poor performance in classifying stage N1 significantly lowers the MF1. From the confusion matrix, we see that the wake stage is most likely to be misclassified as the N1 and REM stages. Stage N1 and REM are rarely misclassified as stage N3, and stage N3 is almost only misclassified as stage N2.

**Figure 5:**
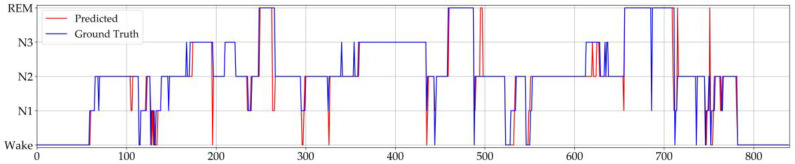
Manually labeled and predicted hypnogram of subject 6 on the first night in the sleep-edfx database.

Figure 5 shows an example of the hypnogram on the first night of subject 6 from the sleep-edfx database. The blue line is the sleep stage manually marked by human experts, and the red line is the model’s prediction. The model has considerable reliability, but it is worth noting that the model is more likely to make mistakes when sleeping transitions from one stage to another. We defined a transition epoch as an epoch whose stage is different from the epoch before or after it, and then counted the data of the first night of subject 6 in the figure below, where the accuracy of nontransition epochs is 96.1%, and the overall accuracy of transition epochs is 57.4%.

### 3.2. Visualization of Attention Weights

**Figure 6:**
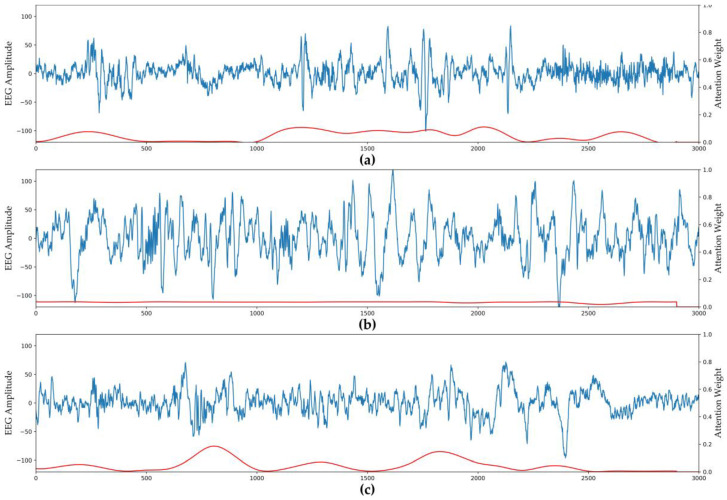
Attention weights visualization of different stages. The blue line is the raw EEG signal and the red line is the corresponding attention weights: (**a**) stage N2; (**b**) stage N3; (**c**) REM stage.

Figure 6 shows that the model uses the attention module to give different weights to different signal regions. Signals with specific characteristics are given more weight, whereas regions with fewer characteristics are given less weight. The blue line in the figure is the raw EEG Fpz-Cz channel signal, and the red line is the weight corresponding to the signal. For the N2 and REM stages (Figure 6a,c), the signal characteristics of different regions in an epoch differ, so the model gives different attention according to the importance of each region. Intuitively, some regions with larger signal amplitudes attract more attention. In stage N3 (Figure 6b), the signal exhibits slow-wave characteristics throughout the epoch, so the model gives almost the same attention to all regions.

### 3.3. Ablation Analysis of Model Components

To explore the effectiveness of each model component, we used the same dataset and training method to train and evaluate different combinations of window feature learning, intra-epoch attention learning, inter-epoch attention learning, and weighted loss-based training. Each combination removed one of the components. When removing window feature learning, the raw window signal was directly used as input to the intra-epoch attention module. When removing the intra- or inter-epoch attention module, the output of the previous module was directly connected to the subsequent GAP layer. Table 5 shows the performance of different combinations. Taking the full model as the baseline, the removal of any component will reduce the model’s MF1 metric. The removal of the window feature caused the greatest decline in performance. After removing the weighted loss function, we found that the model’s accuracy did not decrease, but that the MF1 decreased by 2.0 and 0.4 in the two experiments, indicating that the weighted loss function plays an essential role in improving the model’s accuracy in classifying the minority stage.

### 3.4. Comparison with Other Methods

Table 6 shows a comparison between our work and other methods in terms of overall accuracy, MF1, and per-class F1-score. The comparison is based on experiments on sleep-edfx with subject-wise partitioning and sleep-edf with epoch-wise partitioning. In both cases, our model achieved the best results. In the subject-wise method, our model was better than other methods, except for stages N2 and N3; [26] achieved the highest accuracy on stages N2 and N3, but its F1-score for stage N1 was only 33.2, which is the lowest among all methods. In the epoch-wise method, our model was better than other methods in all metrics. The result shows that our model does not sacrifice the classification accuracy of minority categories to improve the performance of majority categories.

## 4. Discussion

In recent years, many automated sleep stage classification methods based on deep neural networks have used CNNs for feature extraction and vanilla RNNs or LSTM to capture temporal information. These strategies have significantly improved sleep stage classification accuracy. In this study, we used the sliding raw window signal as input to a CNN combined with multiple attention layers as the epoch feature extractor, and used multiple attention layers instead of an RNN structure to ascertain the temporal dependency between epochs. Our method achieved better overall classification accuracy and better performance in minority categories than several state-of-the-art methods. In the feature extraction stage, the CNN module can extract the features of each signal window well. As can be seen from the results of the attention weight visualization component, the attention block can learn that the model should give different attention to different windows based on the importance of each signal window. When an epoch has prominent characteristics, the model should pay more attention to significant areas, and when the characteristics of each signal window in the epoch are relatively similar, the same attention should be given across the epoch. From the results of the module validity analysis, we show that the multiple attention layers can play a role in processing temporal information from multiple epoch inputs, and that the weighted loss function effectively balances the model’s performance on the majority and minority stages.

In the future, we need to do the following work. First, in order to more accurately evaluate the general performance of the automatic sleep staging classification method in actual clinical applications, the model should be tested on additional independent external data, and transfer learning strategy should be applied to improve the generalization of the deep learning model. Second, during manual scoring, human experts combine EEG, EOG, EMG, and other signals to make a comprehensive judgment. However, deep learning-based methods that directly use multichannel data as input have not effectively improved classification accuracy, so we plan to use the attention mechanism employed in this study on multiple channels to improve the model’s classification performance.

## 5. Conclusions

In this study, we proposed a convolution- and attention-based neural network using a single EEG channel to realize automated sleep stage classification. Compared to previous methods, we use the CNN combined with an attention mechanism as a feature extractor, and use multiple attention layers to replace an RNN architecture. The performance of the attention module is consistent with human intuition when classifying sleep stages. Moreover, a weighted loss function played an essential role in solving problems caused by sleep stage class imbalance. Without changing the model architecture and training method, we demonstrate that our model can work well on different databases with different data partitioning methods.

## Figures and Tables

**Table 1:** Number of epochs of each stage in the datasets after processing.

Dataset	W	N1	N2	N3	REM	Total
Sleep-edfx	8246	2804	17,799	5703	7717	42,269
Sleep-edf	8055	604	3621	1299	1609	15,188

**Table 2:** Convolutional layer parameters in the window feature learning component.

Module	Number of Filters	Kernel Size	Stride	Output Shape
Input	-	-	-	(200, 1)
Conv_1	64	5	3	(66, 64)
Conv_2	64	5	3	(21, 64)
Conv_3	128	3	2	(10, 128)
Conv_4	128	3	1	(8, 128)
Conv_5	256	3	1	(6, 256)
GAP	-	-	-	(1, 256)

**Table 3:** Confusion matrix and overall performance on the sleep-edf dataset.

Stage	Predictions					Per-Class Metrics		
	Wake	N1	N2	N3	REM	Precision	Recall	F1
W	2388	33	6	1	5	99.1	98.2	98.6
N1	15	83	25	1	35	52.9	52.2	52.5
N2	2	28	1024	49	16	92.7	91.5	92.1
N3	2	0	44	336	1	86.8	87.7	87.3
REM	2	13	6	0	437	88.5	95.4	91.8

Overall accuracy: 93.7%, MF1 score: 84.5.

**Table 4:** Confusion matrix and overall performance on the sleep-edfx dataset.

Stage	Predictions					Per-Class Metrics		
	W	N1	N2	N3	REM	Precision	Recall	F1
W	7287	586	89	57	149	91.5	89.2	90.3
N1	279	1497	434	24	570	42.1	53.4	47.1
N2	259	846	14,596	1388	710	90.5	82.1	86.0
N3	39	31	586	5042	5	76.6	88.4	82.1
REM	103	598	422	69	6525	82.0	84.6	83.2

Overall accuracy: 82.8%, MF1 score: 77.8.

**Table 5:** Performance of different combinations of model components.

Window Feature	Intra-Epoch Attention	Inter-Epoch Attention	Weighted Loss Function	Overall Performance			
				Subject-Wise		Epoch-Wise	
				Accuracy	MF1	Accuracy	MF1
√	√	√	√	82.8	77.8	93.7	84.5
×	√	√	√	76.7	70.5	83.5	68.2
√	×	√	√	81.3	76.3	92.3	82.2
√	√	×	√	82.0	76.9	93.1	83.7
√	√	√	×	82.8	75.8	**93.8**	84.1

**Table 6:** Performance of different methods on the sleep-edf and sleep-edfx datasets.

	Methods	Samples	Per-Class F1-Score					Overall Performances	
			Wake	N1	N2	N3	REM	Accuracy	MF1
**Sleep-edfx**	Ref. [17]	37,022	71.6	47.0	84.6	84.0	81.4	78.9	73.7
	Ref. [40]	37,022	65.4	43.7	80.6	84.9	74.5	74.9	69.8
	Ref. [29]	41,950	84.7	46.6	85.9	84.8	82.4	82.0	76.9
	Ref. [26]	46,236	89.8	33.2	86.7	86.0	82.6	82.6	74.2
	Proposed	42,269	90.3	47.1	86.0	82.1	83.2	82.8	77.8
**Sleep-edf**	Ref. [19]	15,188	96.9	49.1	88.9	84.2	81.2	90.8	80.1
	Ref. [41]	15,136	97.8	30.4	89.0	85.5	82.5	91.3	77.0
	Ref. [25]	15,188	97.5	24.8	89.4	87.0	80.8	91.2	75.9
	Proposed	15,188	98.6	52.5	92.1	87.2	91.8	93.7	84.5

## References

[1] Bjørnarå K.A., Dietrichs E., Toft M. (2015). Longitudinal Assessment of Probable Rapid Eye Movement Sleep Behaviour Disorder in Parkinson’s Disease. Eur. J. Neurol..

[2] Zhong G., Naismith S.L., Rogers N.L., Lewis S.J.G. (2011). Sleep–Wake Disturbances in Common Neurodegenerative Diseases: A Closer Look at Selected Aspects of the Neural Circuitry. J. Neurol. Sci..

[3] Buysse D.J. (2014). Sleep Health: Can We Define It? Does It Matter?. Sleep.

[4] Wolpert E.A. (1969). A Manual of Standardized Terminology, Techniques and Scoring System for Sleep Stages of Human Subjects. Arch. Gen. Psychiatry.

[5] Iber C., Ancoli-Israel S., Chesson A.L., Quan S.F. (2007). The AASM Manual for the Scoring of Sleep and Associated Events: Rules, Terminology and Technical Specification.

[6] Fiorillo L., Puiatti A., Papandrea M., Ratti P.-L., Favaro P., Roth C., Bargiotas P., Bassetti C.L., Faraci F.D. (2019). Automated Sleep Scoring: A Review of the Latest Approaches. Sleep Med. Rev..

[7] Acharya U.R., Oh S.L., Hagiwara Y., Tan J.H., Adam M., Gertych A., Tan R.S. (2017). A Deep Convolutional Neural Network Model to Classify Heartbeats. Comput. Biol. Med..

[8] Cheng J.-Z., Ni D., Chou Y.-H., Qin J., Tiu C.-M., Chang Y.-C., Huang C.-S., Shen D., Chen C.-M. (2016). Computer-Aided Diagnosis with Deep Learning Architecture: Applications to Breast Lesions in US Images and Pulmonary Nodules in CT Scans. Sci. Rep..

[9] Talo M., Baloglu U.B., Yıldırım Ö., Rajendra Acharya U. (2019). Application of Deep Transfer Learning for Automated Brain Abnormality Classification Using MR Images. Cogn. Syst. Res..

[10] Acharya U.R., Oh S.L., Hagiwara Y., Tan J.H., Adeli H. (2018). Deep Convolutional Neural Network for the Automated Detection and Diagnosis of Seizure Using EEG Signals. Comput. Biol. Med..

[11] Voets M., Møllersen K., Bongo L.A. (2019). Reproduction Study Using Public Data of: Development and Validation of a Deep Learning Algorithm for Detection of Diabetic Retinopathy in Retinal Fundus Photographs. PLoS ONE.

[12] Svetnik V., Ma J., Soper K.A., Doran S., Renger J.J., Deacon S., Koblan K.S. (2007). Evaluation of Automated and Semi-Automated Scoring of Polysomnographic Recordings from a Clinical Trial Using Zolpidem in the Treatment of Insomnia. Sleep.

[13] Macaš M., Grimová N., Gerla V., Lhotská L. (2019). Semi-Automated Sleep EEG Scoring with Active Learning and HMM-Based Deletion of Ambiguous Instances. Proceedings.

[14] Chambon S., Galtier M.N., Arnal P.J., Wainrib G., Gramfort A. (2018). A Deep Learning Architecture for Temporal Sleep Stage Classification Using Multivariate and Multimodal Time Series. IEEE Trans. Neural Syst. Rehabil. Eng..

[15] Sharma M., Goyal D., Achuth P.V., Acharya U.R. (2018). An Accurate Sleep Stages Classification System Using a New Class of Optimally Time-Frequency Localized Three-Band Wavelet Filter Bank. Comput. Biol. Med..

[16] Liang S.-F., Kuo C.-E., Hu Y.-H., Pan Y.-H., Wang Y.-H. (2012). Automatic Stage Scoring of Single-Channel Sleep EEG by Using Multiscale Entropy and Autoregressive Models. IEEE Trans. Instrum. Meas..

[17] Tsinalis O., Matthews P.M., Guo Y. (2016). Automatic Sleep Stage Scoring Using Time-Frequency Analysis and Stacked Sparse Autoencoders. Ann. Biomed. Eng..

[18] Kemp B., Zwinderman A.H., Tuk B., Kamphuisen H.A.C., Oberye J.J.L. (2000). Analysis of a Sleep-Dependent Neuronal Feedback Loop: The Slow-Wave Microcontinuity of the EEG. IEEE Trans. Biomed. Eng..

[19] Hassan A.R., Subasi A. (2017). A Decision Support System for Automated Identification of Sleep Stages from Single-Channel EEG Signals. Knowl.-Based Syst..

[20] Jiang D., Lu Y., Ma Y., Wang Y. (2019). Robust Sleep Stage Classification with Single-Channel EEG Signals Using Multimodal Decomposition and HMM-Based Refinement. Expert Syst. Appl..

[21] LeCun Y., Bengio Y., Hinton G. (2015). Deep Learning. Nature.

[22] Schmidhuber J. (2015). Deep Learning in Neural Networks: An Overview. Neural Netw..

[23] LeCun Y., Boser B., Denker J.S., Henderson D., Howard R.E., Hubbard W., Jackel L.D. (1989). Backpropagation Applied to Handwritten Zip Code Recognition. Neural Comput..

[24] Andreotti F., Phan H., Cooray N., Lo C., Hu M.T.M., De Vos M. Multichannel Sleep Stage Classification and Transfer Learning using Convolutional Neural Networks. Proceedings of the 2018 40th Annual International Conference of the IEEE Engineering in Medicine and Biology Society (EMBC).

[25] Yildirim O., Baloglu U., Acharya U. (2019). A Deep Learning Model for Automated Sleep Stages Classification Using PSG Signals. Int. J. Environ. Res. Public Health.

[26] Phan H., Andreotti F., Cooray N., Chen O.Y., De Vos M. DNN Filter Bank Improves 1-Max Pooling CNN for Single-Channel EEG Automatic Sleep Stage Classification. Proceedings of the 2018 40th Annual International Conference of the IEEE Engineering in Medicine and Biology Society (EMBC).

[27] Hochreiter S., Schmidhuber J. (1997). Long Short-Term Memory. Neural Comput..

[28] Michielli N., Acharya U.R., Molinari F. (2019). Cascaded LSTM Recurrent Neural Network for Automated Sleep Stage Classification Using Single-Channel EEG Signals. Comput. Biol. Med..

[29] Supratak A., Dong H., Wu C., Guo Y. (2017). DeepSleepNet: A Model for Automatic Sleep Stage Scoring Based on Raw Single-Channel EEG. IEEE Trans. Neural Syst. Rehabil. Eng..

[30] Vaswani A., Shazeer N., Parmar N., Uszkoreit J., Jones L., Gomez A.N., Kaiser Ł., Polosukhin I., Guyon I., Luxburg U.V., Bengio S., Wallach H., Fergus R., Vishwanathan S., Garnett R. (2017). Attention Is All You Need. Advances in Neural Information Processing Systems 30.

[31] Goldberger A.L., Amaral L.A., Glass L., Hausdorff J.M., Ivanov P.C., Mark R.G., Mietus J.E., Moody G.B., Peng C.K., Stanley H.E. (2000). PhysioBank, PhysioToolkit, and PhysioNet: Components of a New Research Resource for Complex Physiologic Signals. Circulation.

[32] Szegedy C., Liu W., Jia Y., Sermanet P., Reed S., Anguelov D., Erhan D., Vanhoucke V., Rabinovich A. Going Deeper with Convolutions. Proceedings of the 2015 IEEE Conference on Computer Vision and Pattern Recognition (CVPR).

[33] Ioffe S., Szegedy C. (2015). Batch Normalization: Accelerating Deep Network Training by Reducing Internal Covariate Shift. arXiv.

[34] Vinod N., Geoffrey E.H. Rectified Linear Units Improve Restricted Boltzmann Machines. Proceedings of the 27th International Conference on Machine Learning (ICML-10).

[35] Krizhevsky A., Sutskever I., Hinton G.E., Pereira F., Burges C.J.C., Bottou L., Weinberger K.Q. (2012). ImageNet Classification with Deep Convolutional Neural Networks. Advances in Neural Information Processing Systems 25.

[36] Ba J.L., Kiros J.R., Hinton G.E. (2016). Layer Normalization. arXiv.

[37] Kingma D.P., Ba J. (2017). Adam: A Method for Stochastic Optimization. arXiv.

[38] Zhang M.R., Lucas J., Hinton G., Ba J. (2019). Lookahead Optimizer: K Steps Forward, 1 Step Back. arXiv.

[39] Danker-Hopfe H., Anderer P., Zeitlhofer J., Boeck M., Dorn H., Gruber G., Heller E., Loretz E., Moser D., Parapatics S. (2009). Interrater Reliability for Sleep Scoring According to the Rechtschaffen & Kales and the New AASM Standard. J. Sleep Res..

[40] Tsinalis O., Matthews P.M., Guo Y., Zafeiriou S. (2016). Automatic Sleep Stage Scoring with Single-Channel EEG Using Convolutional Neural Networks. arXiv.

[41] Sharma R., Pachori R.B., Upadhyay A. (2017). Automatic Sleep Stages Classification Based on Iterative Filtering of Electroencephalogram Signals. Neural Comput. Appl..

